# Concordance of gonorrhoea of the rectum, pharynx and urethra in same-sex male partnerships attending a sexual health service in Melbourne, Australia

**DOI:** 10.1186/s12879-018-3003-2

**Published:** 2018-02-27

**Authors:** Vincent J. Cornelisse, Lei Zhang, Matthew Law, Marcus Y. Chen, Catriona S. Bradshaw, Clare Bellhouse, Christopher K. Fairley, Eric P. F. Chow

**Affiliations:** 1 The Melbourne Sexual Health Centre, Alfred Health, 580 Swanston St, Carlton, Melbourne, VIC 3053 Australia; 2 Central Clinical School, Faculty of Medicine, Nursing and Health Sciences, Monash University, Melbourne, Australia; 3 The Kirby Institute, UNSW Sydney, Sydney, Australia

**Keywords:** Gonorrhea [C12.294.668.281.401], Homosexuality [F01.145.802.975.500], Sexual partners [M01.778], Disease transmission, Infectious [N06.850.310], Pharynx [A14.724]

## Abstract

**Background:**

We aimed to describe anatomic site-specific concordance of gonococcal infections in partnerships of men who have sex with men (MSM).

**Methods:**

We conducted a cross-sectional analysis of data from MSM partnerships attending Melbourne Sexual Health Centre between March 2011 and February 2015. Logistic regression models (random effect) were used to examine the association between gonococcal infections of the urethra, rectum and pharynx. Gonococci were detected by culture at all anatomic sites.

**Results:**

The analysis included 495 partnerships. Of the men with urethral gonorrhoea, 33% (95% CI 18–52) had partners with pharyngeal gonorrhoea and 67% (95% CI 48–82) had partners with rectal gonorrhoea. The adjusted odds of having urethral gonorrhoea was 4.6 (95% CI 1.2–17.1) for a man whose partner had pharyngeal gonorrhoea, and 48.1 (95% CI 18.3–126.7) for a man whose partner had rectal gonorrhoea. Of the men with rectal gonorrhoea, 46% (95% CI 31–61) had a partner with urethral gonorrhoea and 23% (95% CI 12–37) had a partner with pharyngeal gonorrhoea. The adjusted odds of having rectal gonorrhoea was 63.9 (95% CI 24.7–165.6) for a man whose partner had urethral gonorrhoea. Of the men with pharyngeal gonorrhoea, 42% (95% CI 23–63) had a partner with rectal gonorrhoea and 23% (95% CI 9–44) had a partner with had a partner with pharyngeal gonorrhoea. The adjusted odds of having pharyngeal gonorrhoea was 8.9 (95% CI 3.2–24.6) for a man whose partner had rectal gonorrhoea. The crude odds of having pharyngeal gonorrhoea was 14.2 (95% CI 5.1–39.0) for a man whose partner had pharyngeal gonorrhoea.

**Conclusions:**

These data provide the first estimates of concordance of anatomic site-specific gonococcal infections in MSM partnerships, and confirm that urethral gonorrhoea is contracted from both rectal and pharyngeal sites, and suggest that gonococci transmit between the rectum and pharynx. However, due to use of culture rather than NAAT, our analysis was not adequately powered to assess pharynx-to-pharynx transmission of gonococci.

## Background

Among populations of men who have sex with men (MSM), infection rates of Neisseria gonorrhoeae (gonococci) have increased worldwide since the early 2000s. [1] In Australia, the rate of gonorrhoea amongst men has doubled in the last 5 years, [2] and most cases are in MSM (68% in 2013). [3] Some of the rise in gonorrhoea diagnoses is likely due to the introduction of nucleic acid amplification tests (NAATs), [4–6] which are more sensitive than culture for rectal and pharyngeal gonococcal infections. [7–9] However, large rises in the true prevalence of infection have occurred in MSM which could not be accounted for by changes in sexual practices. [10] These findings highlight the need to better understand what factors are driving the transmission of gonococci in MSM.

Mathematical models can describe the transmission dynamics of sexually transmissible infections (STIs), and can be used to assess the potential impact of proposed public health interventions. We have previously published a mathematical model for gonococcal transmission among MSM which included multiple anatomic sites. [11] However, no empirical data on gonococcal transmission between sites were available, and our model was difficult to calibrate to data on community prevalence of gonorrhoea. Better mathematical models of transmission require anatomic site-specific gonococcal infection data and estimates of the probability of transmission between anatomic sites.

We sought to gain a clearer understanding of concordance of gonococcal infections between anatomic sites in MSM. With this aim, we analysed a population of MSM who presented for gonorrhoea testing on the same day as their male sexual partner. Previous studies have described the sexual practices associated with gonorrhoea at different anatomic sites, [12, 13] but no study had yet assessed the proportion of gonorrhoea diagnoses at specific anatomic sites in same-sex male partnerships.

## Methods

This was a cross-sectional study of the proportion of gonorrhoea diagnoses at specific anatomic sites in same-sex male partnerships attending Melbourne Sexual Health Centre (MSHC) together on the same day. MSHC is the major public sexual health clinic in Melbourne, Australia. It provides a free walk-in service and no referrals are required. Over the study period MSHC provided approximately 35,000 consultations annually, and about 37% of consultations were for MSM. [14]

### Collection of data

All patients at MSHC completed a computer-assisted self-interview (CASI) that collected their demographic details and details of sexual practices with regular and casual sexual partners, including their number of partners and the use of condoms for peno-anal sex in the last 3 months. [15] CASI asks no questions about oral sex. From March 2011 onwards, the question “Is your partner also being seen today at this clinic?” was added on CASI. Patients who answered “yes” to this question were asked for their partner’s name. We included all MSM who answered “yes” to this question, and whose partner we could identify, and who had testing for gonococci at any anatomic site between March 2011 and February 2015.

Screening for gonorrhoea was conducted according to Australian guidelines, [16] which recommended screening for pharyngeal and rectal gonorrhoea in all MSM regardless of history of potential exposure or symptoms, but the guidelines only recommended testing for urethral gonorrhoea if the patient has symptoms of urethritis. In accordance with the clinical protocol at MSHC, culture methods were used for detection of gonococci at all anatomic sites during the study period. Pharyngeal, rectal and urethral specimens were obtained using cotton-tipped swabs. Rectal specimens were obtained either by blind anal swabbing or, in men with symptomatic proctitis, via anoscopy. Swabs were plated onto modified Thayer-Martin medium for isolation of Neisseria gonorrhoeae (gonococci).

### Statistical analyses

In our statistical analyses we included only couples in which both partners were tested for both rectal and pharyngeal gonorrhoea, as gonococcal infection at these sites is usually asymptomatic [17, 18], and hence including un-tested men would have under-estimated the positivity of gonococcal infections at these anatomic sites.

Condom use data was categorised as “condom use always or no peno-anal sex” versus “condom use not always” in the 3 months before presentation at the clinic, with separate categorisation for contact with regular or casual partners, and for receptive and insertive peno-anal sex.

All statistical analyses were performed using STATA software (version 13.1, StataCorp LP). The 95% confidence intervals (CI) for the proportions of positive results were calculated using ‘exact’ binomial distribution. [19] Multivariate logistic regression was used to calculate the odds of having gonorrhoea at a specific anatomic site, when a partner was diagnosed with gonorrhoea at a specific anatomic site, by clustering the individuals within a couple together. [20] We conducted three separate analyses: (1) men with urethral gonorrhoea treated as the index case, to assess the distribution of gonorrhoea in their partners; (2) men with pharyngeal gonorrhoea treated as the index case, to assess the distribution of gonorrhoea in their partners; and (3) men with rectal gonorrhoea treated as the index case, to assess the distribution of gonorrhoea in their partners. In those partnerships where both partners had gonorrhoea at the same anatomic site, each partner was treated as an index case and a partner. We adjusted for condom use for receptive or insertive peno-anal sex with the regular partner, and for the diagnosis of gonorrhoea at other anatomic sites in the partner.

Ethics approval was obtained from the Alfred Hospital Ethics Committee, Melbourne, Australia (number 108/15).

## Results

During the study period there were 1464 men, in 732 partnerships, who presented together with their partner for testing for gonococci. Of these, 990 men were in 495 partnerships in which both partners had complete testing for rectal and pharyngeal gonorrhoea. These men were included in our analyses, and their demographics are shown in Table 1.

**Table 1:** Demographic and behavioural characteristics of the 990 men who attended the Melbourne Sexual Health Centre with their male partner on the same day and were tested for both anorectal and pharyngeal gonococci by culture

Characteristics	Median (IQR)	Yes/total; %yes
Age (years)	28 (24 to 34)	–
Age difference (years) between partners	4 (2 to 9)	–
HIV positive	–	37/990 (4%)
Consistent condom use for RAI with RSP*	–	350/905 (39%)
Consistent condom use for RAI with CSP*	–	733/894 (82%)
Consistent condom use for IAI with RSP*	–	335/899 (37%)
Consistent condom use for IAI with CSP*	–	716/893 (80%)
Reported one or more CSP for AI in last 3 months	–	440/990 (44%)
Number of CSP in last 3 months for those with CSP.	3 (2 to 5)	–

IQR = interquartile range; RAI = receptive anal intercourse; IAI = insertive anal intercourse; RSP = regular sexual partner; CSP = casual sexual partner

*Consistent condoms use at all times or no anal sex, in the last 3 months

### Site-specific gonorrhoea in the male partners of men with urethral gonorrhoea.

33 men (3.3% of 990, 95% CI 2.3 to 4.6) had urethral gonorrhoea, of whom 11 (33.3%, 95% CI 18.0 to 51.8) had a partner with pharyngeal gonorrhoea and 22 (66.7%, 95% CI 48.2 to 82.0) had a partner with rectal gonorrhoea. In one partnership both men had urethral gonorrhoea (2 of 33 men, or 6.1%, 95% CI 0.7 to 20.2) (Table 2).

**Table 2:** Urethral gonorrhoea in partner 1, and associations with gonococcal infections by anatomic site in partner 2

		P2			% + ve (95% CI)	Crude OR (95% CI)	Adjusted OR^a^ (95% CI)
		Urethra -ve	Urethra +ve	Total			
P1	Urethra -ve	926	31	957	3.2% (2.2 to 4.6)	Ref	Ref
	Urethra +ve	31	2	33	6.1% (0.7 to 20.2)	1.9	0.3
	Total	957	33	990	3.3% (2.3 to 4.6)	(0.4 to 8.4)	(0.1 to 1.8)
		P2			% + ve (95% CI)	Crude OR (95% CI)	Adjusted OR^a^ (95% CI)
		Pharynx -ve	Pharynx +ve	Total			
P1	Urethra -ve	942	15	957	1.6% (0.9 to 2.6)	Ref	Ref
	Urethra +ve	22	11	33	33.3% (18.0 to 51.8)	31.4***	4.6*
	Total	964	26	990	2.6% (1.7 to 3.8)	(13.0 to 76.1)	(1.2 to 17.1)
		P2			% + ve (95% CI)	Crude OR (95% CI)	Adjusted OR^a^ (95% CI)
		Rectum -ve	Rectum +ve	Total			
P1	Urethra -ve	931	26	957	2.7% (1.8 to 4.0)	Ref	Ref
	Urethra +ve	11	22	33	66.7% (48.2 to 82.0)	71.6***	48.1***
	Total	942	48	990	4.8% (3.6 to 6.4)	(31.5 to 162.9)	(18.3 to 126.7)

These analyses include only participants who were tested at both the rectum and pharynx

^a^adjusted for gonorrhoea at other anatomic sites in partner 2, and for consistent condom use for insertive penile-anal sex with partner 2

*p < 0.05

**p < 0.01

***p < 0.001

Abbreviations: P1, “partner 1”; P2, “partner 2”; *CI*, confidence interval; *OR,* odds ratio; aOR

The odds ratio of having urethral gonorrhoea was 4.6 (95% CI 1.2 to 17.1) for a man whose partner had pharyngeal gonorrhoea, and 48.1 (95% CI 18.3 to 126.7) for a man whose partner had rectal gonorrhoea, after adjusting for gonorrhoea at other anatomic sites in the partner, and for consistent use of condoms for insertive peno-anal sex with their regular partner (Table 2).

### Site-specific gonorrhoea in the male partners of men with pharyngeal gonorrhoea

26 men (2.6%, 95% CI 1.7 to 3.8) had pharyngeal gonorrhoea, of whom 11 (42.3%, 95% CI 23.4 to 63.1) had a partner with urethral gonorrhoea and 11 (42.3%, 95% CI 23.4 to 63.1) had a partner with rectal gonorrhoea. In 3 partnerships both men had pharyngeal gonorrhoea (6 of 26 men, or 23.1%, 95% CI 9.0 to 43.6) (Table 3).

**Table 3:** Pharyngeal gonorrhoea in partner 1, and associations with gonococcal infections by anatomic site in partner 2

		P2			% + ve (95% CI)	Crude OR (95% CI)	Adjusted OR^a^ (95% CI)
		Urethra -ve	Urethra +ve	Total			
P1	Pharynx -ve	942	22	964	2.3% (1.4 to 3.4)	Ref	Ref
	Pharynx +ve	15	11	26	42.3% (23.4 to 63.1)	31.4***	18.3***
	Total	957	33	990	3.3% (2.3 to 4.6)	(13.0 to 76.1)	(6.9 to 48.8)
		P2			% + ve (95% CI)	Crude OR (95% CI)	Adjusted OR^a^ (95% CI)
		Pharynx -ve	Pharynx +ve	Total			
P1	Pharynx -ve	944	20	964	2.1% (1.3 to 3.2)	Ref	Ref
	Pharynx +ve	20	6	26	23.1% (9.0 to 43.6)	14.2***	2.5
	Total	964	26	990	2.6% (1.7 to 3.8)	(5.1 to 39.0)	(0.7 to 8.4)
		P2			% + ve (95% CI)	Crude OR (95% CI)	Adjusted OR^a^ (95% CI)
		Rectum -ve	Rectum +ve	Total			
P1	Pharynx -ve	927	37	964	3.8% (2.7 to 5.3)	Ref	Ref
	Pharynx +ve	15	11	26	42.3% (23.4 to 63.1)	18.4***	8.9***
	Total	942	48	990	4.8% (3.6 to 6.4)	(7.9 to 42.8)	(3.2 to 24.6)

These analyses include only participants who were tested at both the rectum and pharynx

^a^ adjusted for gonorrhoea at other anatomic sites in partner 2

*p < 0.05

**p < 0.01

***p < 0.001

Abbreviations: P1, “partner 1”; P2, “partner 2”; *CI,* confidence interval; *OR*, odds ratio; aOR

The odds ratio of having pharyngeal gonorrhoea was 18.3 (95% CI 6.9 to 48.8) for a man whose partner had urethral gonorrhoea and 8.9 (95% CI 3.2 to 24.6) for a man whose partner had rectal gonorrhoea, after adjusting for gonorrhoea at other anatomic sites in the partner. The crude odds ratio of having pharyngeal gonorrhoea was 14.2 (95% CI 5.1 to 39.0) for a man whose partner had pharyngeal gonorrhoea (Table 3).

### Site-specific gonorrhoea in the male partners of men with rectal gonorrhoea

48 men (4.8%, 95% CI 3.6 to 6.4) had rectal gonorrhoea, of whom 22 (45.8%, 95% CI 31.4 to 60.8) had a partner with urethral gonorrhoea, and 11 (22.9%, 95% CI 12.0 to 37.3) had pharyngeal gonorrhoea. In 8 couples both partners had rectal gonorrhoea (16 of 48 men, or 33.3%, 95% CI 20.4 to 48.4) (Table 4).

**Table 4:** Rectal gonorrhoea in partner 1, and associations with gonococcal infections by anatomic site in partner 2

		P2			% + ve (95% CI)	Crude OR (95% CI)	Adjusted OR^a^ (95% CI)
		Urethra -ve	Urethra +ve	Total			
P1	Rectum -ve	931	11	942	1.2% (0.6 to 2.1)	Ref	Ref
	Rectum +ve	26	22	48	45.8% (31.4 to 60.8)	71.6***	63.9***
	Total	957	33	990	3.3% (2.3 to 4.6)	(31.5 to 162.9)	(24.7 to 165.6)
		P2			% + ve (95% CI)	Crude OR (95% CI)	Adjusted OR^a^ (95% CI)
		Pharynx -ve	Pharynx +ve	Total			
P1	Rectum -ve	927	15	942	1.6% (0.9 to 2.6)	Ref	Ref
	Rectum +ve	37	11	48	22.9% (12.0 to 37.3)	18.4***	3.4
	Total	964	26	990	2.6% (1.7 to 3.8)	(7.9 to 42.8)	(1.0 to 12.2)
		P2			% + ve (95% CI)	Crude OR (95% CI)	Adjusted OR^a^ (95% CI)
		Rectum -ve	Rectum +ve	Total			
P1	Rectum -ve	910	32	942	3.4% (2.3 to 4.8)	Ref	Ref
	Rectum +ve	32	16	48	33.3% (20.4 to 48.4)	14.2***	6.9***
	Total	942	48	990	4.8% (3.6 to 6.4)	(7.1 to 28.5)	(2.4 to 20.3)

These analyses include only participants who were tested at both the rectum and pharynx

^a^adjusted for gonorrhoea at other anatomic sites in partner 2, and for consistent condom use for receptive penile-anal sex with partner 2

*p < 0.05

**p < 0.01

***p < 0.001

Abbreviations: P1, “partner 1”; P2, “partner 2”; *CI,* confidence interval; *OR*, odds ratio; aOR

The odds ratio of having rectal gonorrhoea was 63.9 (95% CI 24.7 to 165.6) for a man whose partner had urethral gonorrhoea, and 3.4 (95% CI 1.0 to 12.2) for a man whose partner had pharyngeal gonorrhoea, and 6.9 (95% CI 2.4 to 20.3) for a man whose partner had rectal gonorrhoea, after adjusting for gonorrhoea at other anatomic sites in the partner, and for consistent use of condoms for receptive peno-anal sex with their regular partner (Table 4).

## Discussion

This is the first partner study of concordance of gonococcal infections in MSM couples, and as such is the first study to explore the relative importance of different anatomic sites and transmission routes. The strongest association was seen between urethral and rectal infection, but the association between urethral and pharyngeal infection was also strong. Both these routes of transmission are supported by existing evidence, [21, 22] but the magnitude of the association between partners had not previously been quantified. The association between rectal and pharyngeal infections after adjusting for urethral infections suggests the potential for direct transmission between the pharynx and the rectum.

### Transmission of gonococci to the urethra from the rectum or pharynx

It is likely that most urethral infections presented to our clinic shortly after acquisition of gonorrhoea, as urethral gonorrhoea is usually symptomatic, [23] and it has a short incubation period. [24] It is therefore likely that the majority of men in our study with urethral gonorrhoea had acquired their infection from the rectum or pharynx of their partner, rather than the reverse. Two thirds of men with urethral gonorrhoea had a partner with rectal gonorrhoea and one third of men with urethral gonorrhoea had a partner with pharyngeal gonorrhoea. Given that culture methods are insensitive for the detection of both rectal and particularly pharyngeal gonorrhoea, [7–9] it is likely that our study has substantially underestimated these proportions. It is therefore likely that when MSM present with urethral gonorrhoea, most of their partners will have rectal gonorrhoea and most partners will also have pharyngeal gonorrhoea, and this makes it difficult to determine the relative contribution of each site to urethral infection. Previous research has reported higher bacterial loads of gonorrhoea in the rectum compared to the pharynx suggesting the rectum may be a more infectious site, [25] supporting the suggestion that the rectum is more likely than the pharynx to transmit to the urethra.

### Transmission of gonococci from pharynx to pharynx

We hypothesised that pharynx-to-pharynx transmission may play a significant role in the overall burden of gonorrhoea at a population level. [26, 27] A recent Australian study found that it was possible to culture gonorrhoea in saliva in about 40% of cases of pharyngeal gonorrhoea, and that amongst individuals with culture-positive pharyngeal gonorrhoea, saliva samples were universally positive by NAAT, [28] and men with pharyngeal gonorrhoea have substantial loads of gonococcal DNA in their saliva. [29] Our univariate analysis supports the hypothesis of pharynx-to-pharynx transmission. However, the multivariate model of this association is difficult to interpret due to the small number of partnerships in which both partners had pharyngeal gonorrhoea. Our study design was not ideal to assess pharynx-to-pharynx transmission, given the bias towards urethral cases and the fact that we used relatively insensitive culture rather than NAAT. We were also not able to assess the effect of third-party contact, where both men in a partnership may have had pharyngeal contact with a third party with gonorrhoea. The short duration of pharyngeal gonorrhoea further complicates any inference from this data because the absence of infection may indicate either no transmission, or transmission with natural resolution of infection. The natural untreated duration of gonorrhoea infection of the pharynx lasts perhaps no more than 12 weeks, [30–32] resulting in low prevalence even if its incidence may be quite high.

### Transmission of gonococci between pharynx and rectum

We hypothesised that pharynx-to-rectum transmission contributes to the incidence of rectal gonorrhoea, and this was supported by our univariate analysis. Again, the multivariate model of this association was difficult to interpret due to the small number of pharyngeal infections. Previous studies have shown that oral-anal sexual contact is a risk factor for rectal gonorrhoea, [12] and a plausible mechanism for the transmission of gonococci from pharynx to rectum is supported by the isolation of gonococci in saliva [28] and its common use as a lubricant during peno-anal sex [33, 34]. Alternatively, our findings could also be explained by transmission from the rectum to the pharynx, and indeed men who practice insertive oro-anal sex have previously been shown to be at increased risk of pharyngeal gonorrhoea. [13] This direction of transmission is analogous to rectum-to-pharynx transmission of other bacterial species, as reported for *Shigella spp*. [35]

### Transmission of gonococci from urethra to urethra

We hypothesised that transmission of gonococci from one urethra to another is rare. There was only one partnership in our study where both men had symptomatic urethral gonorrhoea, and both these men also had rectal gonorrhoea providing an alternative source of infection for both men.

### Transmission of gonococci from rectum to rectum

We hypothesised that transmission of gonococci from one rectum to another is rare. In eight partnerships both men tested positive for rectal gonococci, and in multivariate analysis there was an association between rectal infections in one partner with rectal infections in another partner. Of the 16 men in partnerships where both men had rectal gonorrhoea, 5 also had urethral gonorrhoea and 4 also had pharyngeal gonorrhoea, and an additional 1 had both urethral and pharyngeal gonorrhoea. Hence, of the men with rectal gonorrhoea, 6 had partners with only rectal gonorrhoea. It is biologically implausible for rectal gonococcal infections to transmit directly from one rectum to another, and we can only speculate as to the direction of transmission in these partnerships, and these infections may have been due to both partners being infected by partners external to this partnership.

### Limitations

There are some limitations to our study. Firstly, only 68 of the total 732 partnerships had at least one partner with gonorrhoea, and in only 495 partnerships both partners had complete testing for rectal and pharyngeal gonorrhoea, and this has limited the power of our study to detect small associations. However, this is the largest same sex male partnership-based study of gonorrhoea to date. Secondly, it is important to appreciate that of the 68 partnerships with gonorrhoea, 40 (59%) had at least one partner with urethral infection, and all urethral cases were symptomatic. This means that our study is likely to be biased towards cases of recent transmission to the urethra and hence does not reflect true population site-specific incidence. Also, we only tested men for urethral gonorrhoea if they were symptomatic and hence we may have missed asymptomatic urethral infections. However, previous studies have shown that asymptomatic urethral gonorrhoea is rare, [23, 36] hence the omission of asymptomatic urethral screening should not have significantly affected our results. Thirdly, we used culture tests rather than NAAT for detection of gonococci. Culture has the disadvantage of having lower sensitivity at pharyngeal and rectal sites, [7–9, 37–39] and under-detection of rectal and pharyngeal gonococci would have reduced the power of our study to assess concordance of infections within partnerships. Therefore, our findings may have smaller odds ratios and larger confidence intervals than we would have obtained if we had used NAAT. However, the advantage of culture is that positivity is likely to reflect higher gonococcal loads [25] and thus transmissibility, whereas NAAT positivity indicates the presence of gonococcal DNA only, it does not indicate the presence of viable organisms and is more likely to be positive in low-load infections. [25] Low-load gonococcal infections may in theory be less relevant to transmission. [25] Fourthly, we had no data on gonococcal infections in sexual partners other than the partner who presented on the same day, and we cannot exclude that in some of these partnerships both partners may have acquired their infection from a partner outside of the partnership without transmission within the partnership. This limitation could be addressed through phylogenetic analyses of the gonococcal strains found in these partnerships. However, a recent phylogenetic analysis conducted in Melbourne found no significant phylogenetic difference in 33 out of 34 MSM partnerships with concordant gonococcal infections, [40] suggesting either that concordance of gonococcal infections within partnerships is rare in the absence of within-partnership transmission, or alternatively that phylogenetic analyses are not particularly useful to determine whether concordance is due to within-partnership transmission. Fifthly, the definition of “your partner” in CASI is not specified, and we did not collect information on the duration of the relationship, the frequency of sexual contact with that partner, whether their last sexual contact was with that partner, nor whether they engaged in group sex with that partner. Sixthly, we did not collect data on other sexual practices that have been identified as risk factors for gonorrhoea such as frequency and condom use for peno-oral sex, oral-anal contact and the use of saliva as a lubricant for peno-anal sex. [12, 34] Finally, our data did not record detailed information on number of acts, hence we cannot calculate per-act transmission probabilities.

## Conclusions

This study confirms that symptomatic urethral gonorrhoea is contracted from both rectal and pharyngeal sites, and suggests that gonococci transmit between pharynx and rectum. It is possible that transmission from pharynx-to-pharynx occurs also, but we were unable to assess this association with certainty. Notwithstanding the limitations of our study, these findings provide an indication of the strength of association between gonococcal infections of the rectum, pharynx and urethra in same-sex male couples, which may be used to infer transmissibility. Hence these data may be useful in the development of mathematical models to assess public health interventions to address the rising rates of gonorrhoea in MSM [1]. With the current emphasis on biomedical HIV prevention strategies, such as pre-exposure prophylaxis and “treatment as prevention”, we will likely need to find additional public health control strategies for bacterial sexually transmitted infection that rely less on the promotion of condom use for peno-anal sex. Additional strategies that are currently being investigated include doxycycline prophylaxis for syphilis and chlamydia, [41] and oropharyngeal rinses (mouthwash) for pharyngeal gonorrhoea. [27, 42, 43] Also, several current STI screening guidelines, including those in the US, advise to screen for rectal and pharyngeal gonorrhoea if the patient reports a history of potential exposure at these anatomic sites in the form of an inserted penis. Our data suggest that there may be transmission between rectum and pharynx, which suggests that the definition of exposure is not limited to the insertion of a penis. The exact exposure that should prompt rectal screening has not been accurately defined but may relate to ‘saliva’ exposure and ‘any’ insertion including fingers; but at this stage it may be premature to define this. Perhaps a workable approach may be to offer screening for pharyngeal and rectal gonorrhoea to all sexually active MSM, regardless of a history of site-specific exposure through insertion of a penis, as is recommended by the current Australian guidelines. [16]

## Abbreviations

CASI: Computer-assisted self-interview
CI: Confidence interval
CSP: Casual sexual partner
DNA: Deoxyribonucleic acid
HIV: Human immunodeficiency virus
IAI: Insertive anal intercourse
MSHC: Melbourne Sexual Health Centre
MSM: Men who have sex with men
NAAT: Nucleic acid amplification test
OR: Odds ratio
RAI: Receptive anal intercourse
RSP: Regular sexual partner
STI: Sexually transmissible infections
